# Preparation and In Vitro/In Vivo Characterization of Mixed-Micelles-Loaded Dissolving Microneedles for Sustained Release of Indomethacin

**DOI:** 10.3390/pharmaceutics16121505

**Published:** 2024-11-22

**Authors:** Baojie Wang, Langkun Liao, Huihui Liang, Jiaxin Chen, Yuqin Qiu

**Affiliations:** 1 The Third People’s Hospital of Longgang District, Shenzhen 518112, China; wangbaojie202410@163.com; 2 Department of Pharmaceutics, School of Pharmacy, Guangdong Pharmaceutical University, Guangzhou Higher Education Mega Center, 280 East Waihuan Road, Guangzhou 510006, China; liaolk2019@163.com (L.L.); lianghh202111@163.com (H.L.); chenjiaxin201903@163.com (J.C.); 3 Guangdong Provincial Key Laboratory for Research and Evaluation of Pharmaceutical Preparations, Guangdong Provincial Engineering Center of Topical Precise Drug Delivery System, Guangdong Pharmaceutical University, Guangzhou 510006, China

**Keywords:** indomethacin, mixed micelles, sustained release, adjuvant induced arthritis, dissolving microneedles

## Abstract

**Background/Objectives:** Indomethacin (IDM) is commonly used to treat chronic inflammatory diseases such as rheumatoid arthritis and osteoarthritis. However, long-term oral IDM treatment can harm the gastrointestinal tract. This study presents a design for encapsulating IDM within mixed micelles (MMs)-loaded dissolving microneedles (DMNs) to improve and sustain transdermal drug delivery. **Methods:** Indomethacin-loaded mixed micelles (IDM-MMs) were prepared from Soluplus^®^ and Poloxamer F127 by means of a thin-film hydration method. The MMs-loaded DMNs were fabricated using a two-step molding method and evaluated for storage stability, insertion ability, in vitro release, in vitro transdermal penetration, and in vivo PK/PD studies. **Results:** The obtained MMs were stable at 4 °C and 30 °C for 60 days. The in vitro IDM transdermal penetration was remarkably improved by the MMs-loaded DMNs compared to a commercial patch. A pharmacokinetic study demonstrated that the MMs-loaded DMNs had a relative bioavailability of 4.1 in comparison with the commercial patch. Furthermore, the MMs-loaded DMNs showed a significantly shorter lag time than the commercial patch, as well as a more stable plasma concentration than the DMNs without MMs. The therapeutic efficacy of the IDM DMNs was examined in Complete Freund’s Adjuvant-induced arthritis mice. The IDM DMN treatment effectively reduced arthritis severity, resulting in decreased paw swelling, arthritis index, spleen hyperplasia, and serum IL-1β and TNF-α levels. **Conclusions:** Our findings demonstrated that the novel MMs-loaded DMNs were an effective strategy for sustained IDM release, providing an alternate route of anti-inflammatory drug delivery.

## 1. Introduction

Indomethacin (IDM), a non-steroidal anti-inflammatory drug (NSAID), is widely used for the treatment of chronic inflammatory diseases such as rheumatoid arthritis and osteoarthritis. Oral administration of IDM is very useful, but like other NSAIDs, long-term use of IDM is often associated with gastrointestinal (GI) tract complications, including bleeding, ulcerations, and potential perforation of the stomach or intestines, which can be life-threatening [1,2]. Additionally, IDM has a narrow therapeutic window and requires frequent dosing [3,4]. In view of these problems, it is important to design an efficient drug delivery system to reduce toxicity to the GI tract and to provide a sustained release of IDM.

The topical administration of NSAIDs offers numerous advantages over oral administration, including a minimization of local damage to the GI tract, continuous drug delivery, and avoidance of the hepatic first-pass effect [5,6]. However, the stratum corneum serves as a formidable barrier to percutaneous drug absorption. IDM is classified as a Class II drug, characterized by poor water solubility, which complicates its transdermal delivery. The bioavailability of IDM following topical application is typically less than 5% compared to equivalent oral doses [7,8]. Although several IDM patches have been commercialized, the incorporation of chemical penetration enhancers in these patches can lead to skin irritation and adverse reactions [9,10]. Therefore, specialized formulation techniques are necessary to improve the aqueous solubility of IDM and achieve an adequate transdermal IDM dose.

Recently, more attention has focused on microneedles (MNs) for transdermal drug delivery [11,12,13,14]. MNs typically consist of multiple micron-scale needles of 50~1000 μm in height, which can penetrate the stratum corneum and deliver drugs into the dermis, thereby increasing permeability. Their short length allows for the minimal stimulation of nerve endings, resulting in a relatively painless and minimally invasive drug delivery method [15]. Dissolving microneedles (DMNs), made from water-soluble polymers [16,17], offer distinct advantages over other types of MNs, including the elimination of biohazardous sharps waste, ease of fabrication, and relatively high drug loading capacity. DMNs containing diclofenac sodium were fabricated by Silva et al. [18]. After 40 min of dissolution in PBS, approximately 98% of the incorporated drug was released from the diclofenac-sodium-loaded MNs. The authors stated that DMNs may be a promising platform for the transdermal delivery of NSAIDs. However, the rapid dissolution of MN tips in interstitial fluid often leads to a burst release of the drug, resulting in frequent dosing and poor patient compliance.

Consequently, nanocarrier-loaded DMNs for prolonged therapeutic effect have been under investigation for chronic conditions [19,20,21,22]. Li et al. developed diclofenac-nanoparticle-loaded MNs using a novel nano-in-micro technique [23]. The nanoparticle-loaded MNs demonstrated rapid drug uptake and extended release, sustaining drug levels in tissues for up to 72 h, according to in vivo studies on rats. Nanocarriers can be used to sustain drug release and improve the unfavorable solubility of hydrophobic drugs. In order to deliver IDM to cancer cells, Thiruchenthooran et al. prepared IDM-loaded nanostructured lipid carriers. The findings indicate that IDM with lipid nanoparticle encapsulation has strong anticancer potential [24]. IDM was loaded into small molecules modified mesoporous silica nanoparticles, which were able to form hydrogen bonds with drugs and convert the drugs’ crystal phase into an amorphous state, improving the drugs’ solubility when compared to the raw drug [25]. Recently, amphiphilic-block-copolymer-based mixed micelles (MMs) system showed superior characteristics such as high drug-loading capacity and excellent thermodynamic stability [26,27,28,29].

In the present study, we aimed to achieve effective and continuous transdermal delivery of IDM through the utilization of DMNs in combination with MMs. Firstly, IDM was formulated with Soluplus^®^ and Poloxamer F127 (PF127) to create novel IDM-loaded MMs. The resulting IDM-MMs were characterized in terms of morphology, particle size, stability, and release kinetics. Afterwards, DMNs loaded with IDM-MMs were prepared, followed by the characterization of their mechanical properties, drug loading capacity, and in vitro skin penetration. Furthermore, the pharmacokinetics of IDM following DMN application were evaluated in rats. Finally, the therapeutic efficacy of IDM DMNs in mitigating inflammation in a mouse model of adjuvant-induced arthritis (AIA) was investigated.

## 2. Materials and Methods

### 2.1. Materials

Soluplus^®^ was kindly gifted by BASF (Ludwigshafen, Germany). PF127 and IDM (purity > 98%) were purchased from Shanghai Macklin Biochemical Co., Ltd. (Shanghai, China). PVP/VA S-630 was obtained from Ashland Co., Ltd. (China). Acetonitrile and methanol were purchased from Dikma Technologies, Inc. (Beijing, China). All other reagents were of analytical grade and obtained from standard commercial suppliers.

Male Kunming mice weighing 20 ± 2 g and male Sprague–Dawley (SD) rats weighing 200 ± 20 g were purchased from the laboratory animal center of Southern Medical University (Guangdong, China) and were maintained under standard conditions. The research adhered to the National Institute of Health’s Guide for the Care and Use of Laboratory Animals, eighth edition. All procedures complied with the National Institute of Health and Nutrition Guidelines for the ethical use of animals and received approval from the Experimental Animal Center of Guangdong Pharmaceutical University.

### 2.2. Preparation of Mixed Micelles

The IDM-loaded mixed micelles (MMs) were prepared using a thin-film hydration method, as previously described, with minor modifications [30,31]. In brief, IDM and carrier materials (1:10, *w*/*w*) composed of Soluplus^®^ and PF127 (4:1, *w*/*w*) were dissolved in ethanol within a clean round bottom flask. Ethanol was evaporated under reduced pressure by rotary evaporation to form a thin film. To eliminate residual ethanol, the film was maintained in a vacuum for 24 h at room temperature. After the drying procedure, the film was hydrated with a 2.5% sucrose solution and stirred until complete dissolution of the thin film was achieved. A probe sonicator was employed to sonicate the dispersion for 30 min in an ice bath, equipped with a 5 mm tapered micro-tip probe at 30% amplitude. Finally, the dispersion was filtered through a 0.22 μm filter to remove unincorporated drug, followed by lyophilization.

### 2.3. Characterization of IDM-MMs

#### 2.3.1. Visualization of IDM-MMs

The morphology of IDM-MMs was observed using a transmission electron microscope (TEM, Hitachi, Ltd., Tokyo, Japan). A drop of the IDM-MMs was deposited onto a copper grid and negative-stained with a phosphotungstic acid solution (2%, *w*/*v*).

#### 2.3.2. Particle Size and Zeta Potential

The average particle size, polydispersity index (PDI), and zeta potential of the IDM-MMs were measured by dynamic light scattering (DLS) and phase analysis light scattering (PALS) method (Zetasizer Nano S, Malvern, UK). Each sample was diluted to an appropriate concentration prior to measurement. Every sample was tested three times using a medium stable count rate in a multimodal mode.

#### 2.3.3. Encapsulation Efficiency and Drug Loading

The determination of encapsulation efficiencies (EE%), as well as drug loading coefficient (DL%), was carried out using dynamic dialysis method [32]. In short, to separate free drug from IDM-MMs, a dialysis bag (molecular weight cutoff 3500) containing 1mL of IDM-MMs was sealed and immersed into 100 mL of PBS (pH7.4) at 25 °C with a shaker oscillating at 100 rpm. Samples of 3 mL were withdrawn at predetermined time intervals (10, 20, 30, 40, 60, and 120 min) and same volume of fresh PBS was replaced immediately.

IDM concentration was quantified using a high-performance liquid chromatography (HPLC) system (LC-2010, Shimadzu, Japan). An ODS C18 column (250 × 4.6 mm, 5 μm, Dikma technology, Beijing, China) was used with the mobile phase having a flow rate of 1.0 mL/min. The column temperature was set at 30 °C. Acetonitrile-0.1 mol/L ethylic acid in water (70:30 *v*/*v*) was the chromatographic condition employed. Detection of IDM was performed at a wavelength of 320 nm.

Following the calculation of drug concentrations at each time interval, a drug-concentration-versus-time profile was generated, from which the time of dialysis equilibrium was determined based on the inflection point of the curve. Using this inflection point, the EE% and DL% values were calculated according to the following equations:

(1) $$EE(\%)=1-\frac{amount\;of\;free\;IDM}{amount\;of\;added\;IDM}\times 100\%$$

(2) $$DL\left(\%\right)=\frac{amount\;of\;IDM\;in\;micelles}{amount\;of\;the\;added\;polymer\;and\;IDM}\times 100\%$$

### 2.4. Fabrication of IDM-Loaded and IDM-MMs-Loaded DMNs

The PDMS mold was fabricated as previously described by Lee at al., with slight modifications [33]. A polymethylmethacrylate (PMMA) grid was made by the Technical Institute of Physics and Chemistry, Chinese Academy of Sciences (Beijing, China). The grid had a steel microneedle array glued on it. The steel microneedle array covered a 10 × 10 mm^2^ surface with 400 (20 × 20) microneedles that were 550 µm high. A master structure was created by combining the grid with the steel microneedle array. A PDMS mold was prepared from this master structure. Briefly, a Sylgard 184-base silicone elastomer and a curing agent were mixed at a 10:1 weight ratio and then poured into the master structure. The PDMS mold was cured overnight by baking the MN master with the mixture at 80 °C. After it was completely cured, the PDMS mold was taken off the MN master.

**Figure 1:**
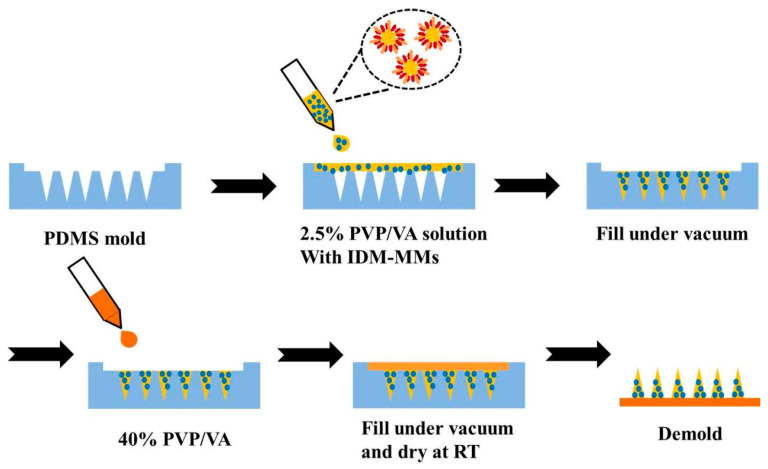
Schematic illustration of the fabrication of IDM-MMs-loaded DMNs.

A two-step molding method was utilized to prepare IDM- or IDM-MMs-loaded DMNs. The process is illustrated schematically in Figure 1. First, a predetermined quantity of IDM and PVP/VA was dissolved in ethanol to create a solution designated for the preparation of the needle tips of IDM-loaded DMNs, referred to as the tip solution. A suitable amount of lyophilized IDM-MMs was dispersed in a PVP/VA aqueous solution, and the mixture served as the tip solution for IDM-MMs-loaded DMNs. The concentration of PVP/VA in both tip solutions was maintained at 2.5% *w*/*w*. For the fabrication of the DMN tips, 45 μL of the respective tip solutions was applied to the PDMS mold and subjected to vacuum for 10 min at room temperature. Next, 200 μL of a 40% *w*/*w* PVP/VA aqueous solution was added to the mold and vacuumed for another 10 min to form a backing layer. Finally, the mold was placed in a desiccator at room temperature for 24 h prior to the separation of the DMNs from the PDMS molds.

The IDM concentration in the tip solutions was set to 8 mg/mL to facilitate the detection of IDM in samples for in vitro skin penetration studies, resulting in a loading of 360 μg of IDM in each piece of IDM or IDM-MMs-loaded DMNs. To achieve a 6 mg/kg dosage for each mouse in pharmacodynamic (PD) studies, a reduced IDM concentration of 2.7 mg/mL was employed in the tip solution, yielding 120 μg of IDM per IDM or IDM-MMs-loaded DMNs.

### 2.5. Characterization of IDM-MMs-Loaded DMNs

The morphology of the IDM-MMs-loaded DMNs was visually examined using a bio-microscope (XSP-8CA, Shanghai optical instrument factory, Shanghai, China) and a scanning electron microscope (EVO MA 10, Carl Zeiss, Oberkochen, Germany).

### 2.6. Stability Studies

Stability studies were conducted to assess the stability of the IDM-MMs under various environmental conditions. The IDM-MMs were lyophilized and stored at 30 ± 2 °C/65 ± 5% relative humidity (RH) and at 4 ± 2 °C for 2 months. The samples were withdrawn at specified time points (0, 1, 5, 10, 20, 30 and 60 days). During the storage period, particle size, polydispersity index (PDI), and encapsulation efficiency (EE%) were monitored at designated intervals.

Furthermore, the stability of the IDM-MM-loaded DMNs was investigated. IDM-MMs DMNs were placed in sealed aluminum foil bags. Each bag contained 5 pieces of DMN, which were stored at ambient temperature (25 °C) or in a refrigerator (4 °C) and taken out at predetermined intervals (1, 5, 10, 30 days). The DMN tips were scraped off with a blade, collected in a vial, and dissolved in a specific amount of water. Particle size and PDI of the IDM-MMs in the redissolved suspension were measured.

### 2.7. Insertion Studies of DMNs

In order to evaluate the insertion properties of the DMNs, a commercial polymeric film (Parafilm^®^ M film) was used as a skin simulant with the method developed and validated previously [12,34,35]. Briefly, an artificial skin model was obtained by six layers of Parafilm^®^ M (~750 µm). Next, the DMN array was inserted into the skin model using a force of 40 N for 30 s after attaching to the insertion applicator. The DMNs were then detached from the Parafilm^®^ M layer. Subsequently, the number of holes created in each Parafilm^®^ M layer was examined microscopically.

Additionally, rat skin was used to test the DMNs’ insertion ability in vitro. For 30 s, a piece of MN patch was pressed on the rat skin with 40 N of force. The treated region was cleaned with a cotton swab following the removal of the MNs from the skin. After treatment with MNs, the skin was fixed for more than 24 h in 4% paraformaldehyde. The skin was then embedded in paraffin, sliced longitudinally, and stained with hematoxylin–eosin (H&E). Micropores in skin slices were observed using a microscope (EVO MA 10, Carl Zeiss, Germany).

### 2.8. In Vitro Release of IDM from MMs and the DMNs

To generate a saturated IDM solution, IDM was dispersed in PBS (pH 7.4) and stirred for 24 h at 37 °C. Excess IDM crystals were present to assure the same dose with the micellar formulation. The in vitro release of IDM from MMs compared with saturated IDM solution was investigated by dialysis method, which was similar to those described in Section 2.3.3 [32]. Briefly, 1 mL of IDM-MMs or saturated IDM solution containing 1 mg of IDM was placed into a dialysis bag. Next, the end-sealed dialysis bag was completely submerged in 100 mL of release medium at 37 °C. This medium was constantly shaken at 100 rpm for 24 h. At intervals of 0.5, 1, 2, 4, 8, 12, and 24 h, sampling aliquots of 3 mL were taken out and replaced with an equivalent volume of new medium. After filtering through a 0.22 µm membrane filter, the sample aliquots were subjected to HPLC analysis.

The in vitro release of IDM from the IDM DMNs and IDM-MMs-loaded DMNs was carried out using the dialysis method, which was subjected to an identical process to the in vitro release from MMs, with minor differences. In this portion, a piece of DMN was put in the dialysis bag rather than 1 mL of IDM-MMs or IDM solution.

### 2.9. In Vitro Transdermal Delivery of IDM from DMNs

The penetration of IDM from DMN arrays across dermatomed porcine ear skin (1 mm) was evaluated using modified Franz diffusion cells, as described previously [12]. Firstly, the drug-loaded DMNs were placed on top of the pre-equilibrated porcine skin and inserted with an applicator at a force of 40 N for 30 s. Next, with the DMNs fixed, the treated porcine skin was attached to a receptor cell containing a stirrer. The donor cells were mounted onto the receptor cells sealed by Parafilm^®^ M to prevent moisture volatilization. The receptor cells were filled with 20% PEG400-PBS as receptor medium. The receptor cell was thermo-regulated at 37 °C and stirred at 300 rpm to create sink conditions. As the control, a commercial IDM patch (0.5 mg/cm^2^, Kowa Co., Ltd., Tokyo, Japan) was applied to the donor cell. The dose of IDM in each piece of DMN or patch was 360 μg. The Franz cell contents were removed at appointed time points and an equal volume of pre-warmed receptor medium was subsequently added to replace this.

The determination of skin retention of IDM after transdermal penetration study was conducted by extracting the drug from porcine skin. In short, the porcine skin, after being washed three times to remove any residual formations and mediums, was chipped into pieces using scalpel and was placed into a grinder. To extract IDM from skin, 2 mL methanol was added into the grinder and the mixture was homogenized for 10 min. The sample was then centrifuged for 15 min (4000 rpm), supernatants were collected and concentrated by nitrogen, and the amount of IDM was detected by HPLC. The experiments were conducted in triplicate.

### 2.10. In Vivo Percutaneous Absorption of IDM DMNs and IDM Patch

Healthy, male Sprague–Dawley rats weighing 200 ± 20 g were housed in animal center for at least one week before the experiment to accommodate the new environment. Rats were randomly divided into three groups, each containing six rats, and were anaesthetized by intraperitoneal injection of 20% phenobarbital sodium. Hair in dorsal skin was carefully shaved 12 h before the experiments. In the DMN groups, IDM-MMs DMNs or IDM DMNs were inserted into the back of each rat with an applicator at a force of 40 N for 30 s. In the control group, a commercial patch was applied to each rat. The doses of IDM for IDM-MMs DMNs, IDM DMNs, and commercial patch were 720 μg, 400 μg, and 2000 μg, respectively. The doses in IDM-MMs DMNs and commercial patch were higher than those of IDM DMNs for the detection of IDM in plasma by HPLC. All the patches applied to the rats were kept in place for 12 h with Microfoam™ Surgical Tape (3 M, Bracknell, UK). At pre-determined time, blood samples were collected from angularis oculi vein into heparin-anticoagulated polyethylene tubes. The plasma was obtained by centrifugation and stored at −20 °C for further detection.

Ethyl acetate was used to extract IDM from plasma samples. Initially, 20 μL of internal standard solution (ethylparaben solution 400 μg/mL in methanol) was added to 200 μL of plasma samples and blended for 30 s, and then a volume of 1 mL ethyl acetate was added and vortexed for 4 min to extract IDM. After centrifugation at 8000 rpm for 10 min, 800 μL of supernatant was collected into a clean tube, and the ethyl acetate was evaporated under nitrogen. The residual was re-dissolved in 100 μL methanol and centrifuged prior to HPLC analysis.

The pharmacokinetic parameters were calculated by DAS 2.0. The relative plasma bioavailability (Frel) values of IDM-MMs DMNs and IDM DMNs compared to IDM patch were calculated using the following equation:

(3) $$Frel=\frac{{AUC}_{DMNs}\cdot {Dose}_{patch}}{{AUC}_{patch}\cdot {Dose}_{DMNs}}$$

### 2.11. Anti-Inflammatory Efficacy of IDM-MMs-Loaded DMNs

#### 2.11.1. Adjuvant-Induced Arthritis in Mice and Administration

Male Kunming mice weighing 20 ± 2 g were given 7 days to acclimate to the laboratory setting before the experiment. The mice were randomly divided into four groups (each including six mice): Group I, negative control group; Group II, adjuvant-induced arthritis (AIA, model group); Group III received a commercial IDM patch (patch group); Group IV received IDM-MMs-loaded DMNs (DMNs group). The adjuvant-induced arthritis (AIA) model was induced in mice on day 0 of the experiment, as previously described, with slight modifications [36]. Mice were given a subcutaneous injection of 100 µL of complete Freund’s adjuvant (CFA), which contained 1 mg of dry, heat-killed mycobacterium tuberculosis (strain H37Ra) per 1.0 mL sterile, non-metabolizable oils (Sigma-Aldrich, St. Louis, Mo, USA) in the plantar area of their right hind paw. The control group received an equivalent volume of saline. IDM administration began on day 7, and hair in abdominal skin of mice was carefully removed 24 h before the therapy. In the patch group, a commercial patch (0.5 mg/cm^2^, Kowa Co., Ltd., Tokyo, Japan) was applied to the mice’s abdomen skin on a daily basis. IDM-MMs-loaded DMNs were manually inserted into the skin of the mice’s abdomens every 2 days in the DMNs group. In the patch group, the administration took place once a day since, according to pharmacokinetic investigations, it was not possible to detect plasma IDM after 48 h at a significantly higher dose than in the DMNs group. The IDM-MMs-loaded DMNs group demonstrated sustained release within 48 h and was treated every two days to prevent skin irritation. Commercial patches and DMNs were withdrawn 12 h after administration. Before reapplying, the leftover patches and DMNs were removed with saline-soaked cotton swabs. The IDM treatment lasted for 7 days (7 times for patch group and 4 times for DMNs group). Both the patch and DMNs group received 6 mg/kg of IDM per dosage. The mice from the negative control and AIA model groups received no therapy.

#### 2.11.2. Assessment of Joint Arthritis

To assess the anti-inflammatory effect during the development of AIA, the circumference of the right hind paw was measured before and 24 h after the adjuvant injection, as well as daily during the experiment. The swelling ratio was calculated using the following equation: swelling ratio (%) =$\;\frac{C_{t}\;-\;C_{0}}{C_{0}}\times 100\%$, where C_0_ is the circumference of the right hind paw at the zero moment (before the injection of CFA), and C_t_ is the circumference of right hind paw on day t.

The mice were observed every day for the severity of joint inflammation until sacrificed. The severity of arthritis was scored on a 0–4 scale [37,38]: 0 = no edema or swelling, 1 = slight edema and limited erythema, 2 = slight edema and erythema from the ankle to the tarsal bone, 3 = moderate edema and erythema from the ankle to the tarsal bone, and 4 = edema and erythema from the ankle to the entire leg. The arthritis score for each mouse was the sum of the scores for all 4 paws.

#### 2.11.3. Serum IL-1β and TNF-α Assay

Blood was collected from the mice’s retro-orbital veins at the end of the experiment (day 14), and serum was obtained by centrifugation at 3000 rpm for 15 min at 4 °C. Serum levels of IL-1β and TNF-α were measured using an enzyme-linked immunosorbent assay (ELISA, Ruixin Biotechnology Co., Ltd., Quanzhou, China), according to manufacturer instructions.

#### 2.11.4. Spleen Index Determination

The mice in all the groups were sacrificed at day 14 via anesthesia. The spleen was promptly dissected and weighed. The spleen index was calculated as the ratio (mg/g) of spleen wet weight to the body weight [37].

### 2.12. Statistical Analysis

All experiments were repeated at least three times and data were presented as mean value ± standard deviation (SD). Data were subjected to one-way analysis of variance (ANOVA) with the aid of SPSS (version 17.0). The threshold for significance was *p* < 0.05.

## 3. Results and Discussions

### 3.1. Characterizations of IDM-MMs

**Figure 2:**
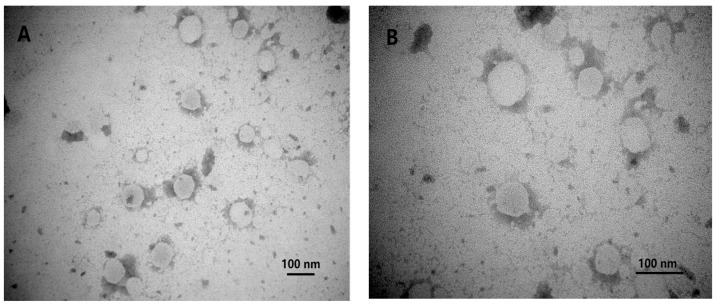
TEM image of IDM-MMs (**A**). TEM image of IDM-MMs (close-up) (**B**).

The morphological features of the IDM-MMs observed by TEM are shown in Figure 2. The IDM-MMs exhibited a spherical morphology. It was determined by DLS that the average particle size of the IDM-MMs was 66.2 ± 0.929 nm, with a PDI of 0.100 ± 0.015, indicating a narrow size distribution. These findings were consistent with the results obtained from the TEM. The zeta potential of the IDM-MMs was −8.72 ± 0.56 mV. The DL% and EE% of the obtained IDM-MMs were 5.04 ± 0.20% and 80.43 ± 4.52%, respectively. Following lyophilization, the MMs exhibited a particle size of 69.2 ± 1.069 nm, a PDI of 0.125 ± 0.035, and an EE% of 81.39 ± 1.67%. No significant differences were observed in particle size, PDI, or EE% post-lyophilization.

### 3.2. Characterization of IDM-MMs DMNs

**Figure 3:**
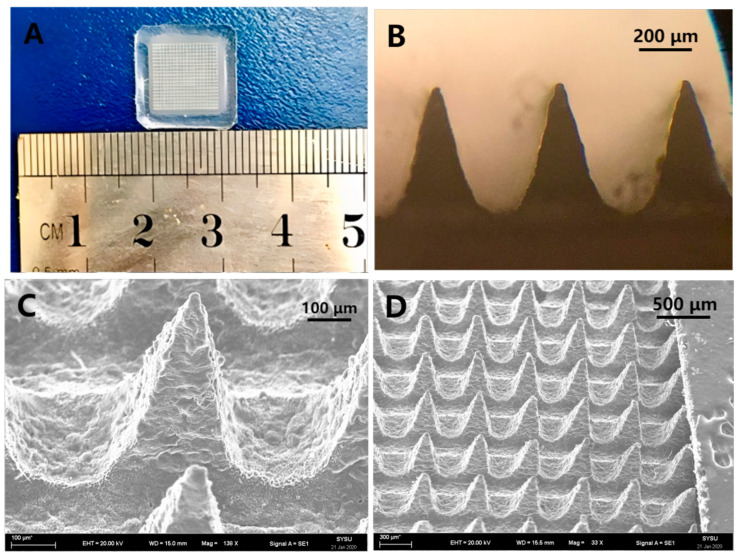
The morphology of IDM-MMs-loaded DMNs. Photograph of IDM-MMs-loaded DMNs (**A**); microscope image of the tips of IDM-MMs-loaded DMNs (**B**); SEM image of IDM-MMs-loaded DMNs (close-up) (**C**); SEM image of IDM-MMs-loaded DMNs (**D**).

Figure 3A,B shows the appearance of IDM-MMs-loaded DMNs imaged using bio-microscope. It was an opaque, square patch containing 400 needles in a 1 cm^2^ area. Figure 3B shows the specific geometric shape and dimension parameters measured by Image J software (v1.8.0.345). Figure 3C,D shows the morphology of the IDM-MMs DMNs using SEM. Each needle in the DMNs was in the shape of a pyramid with a height of 504 μm and a base width of 260 μm.

### 3.3. Stability Studies

**Figure 4:**
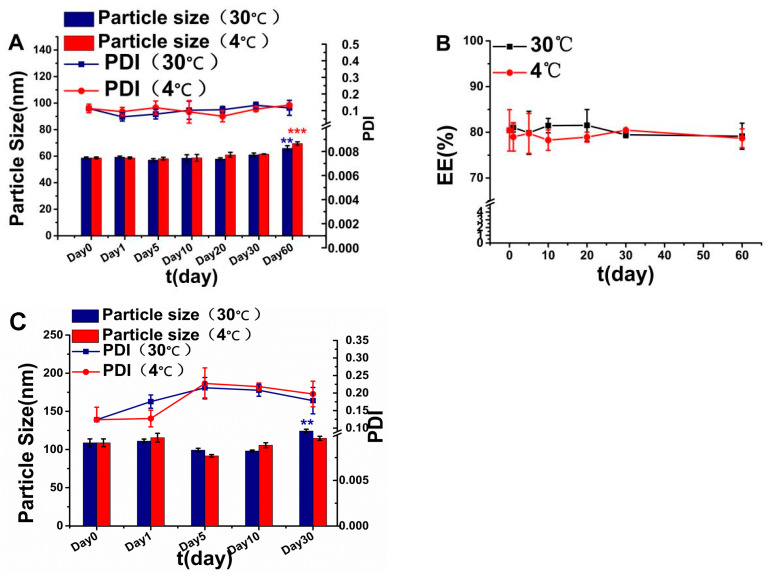
The storage stability of IDM-MMs and IDM-MMs-loaded DMNs. Changes in particle size and PDI of IDM-MMs (**A**); changes in EE% of IDM-MMs (**B**); changes in particle size of IDM-MMs redissolved from the IDM-MMs-loaded DMNs (**C**) (mean ± SD, n = 3) (** *p* < 0.01 compared with day 0, *** *p* < 0.001 compared with day 0).

The stability of IDM-MMs under storage conditions is presented in Figure 4A,B. There was no significant difference in particle size, PDI or EE after storage for 30 days either at 4 °C or at 30 °C. Although the particle size increased by approximately 10 nm after storage for 60 days, there was no significant change in PDI and EE%, indicating no significant aggregation or drug leakage from the IDM-MMs during storage.

After mixing with PVP/VA, filling PDMS molds, and drying, the IDM-MMs were loaded into DMNs and stored. Each of these processes could have an effect on the stability of the IDM-MMs. As shown in Figure 4C, the average particle size of the IDM-MMs increased from 58 nm to 110 nm after loading into DMNs. At the same time, the PDI of the IDM-MMs did not exceed 0.30, indicating that, although they are slightly aggregated, the IDM-MMs maintain good dispersion in the redissolved solution. The particle size of MMs changed considerably (*p* < 0.01) on the 30th day compared to the 0th day when stored at room temperature. No significant changes were observed at other time points.

### 3.4. Insertion Studies

**Figure 5:**
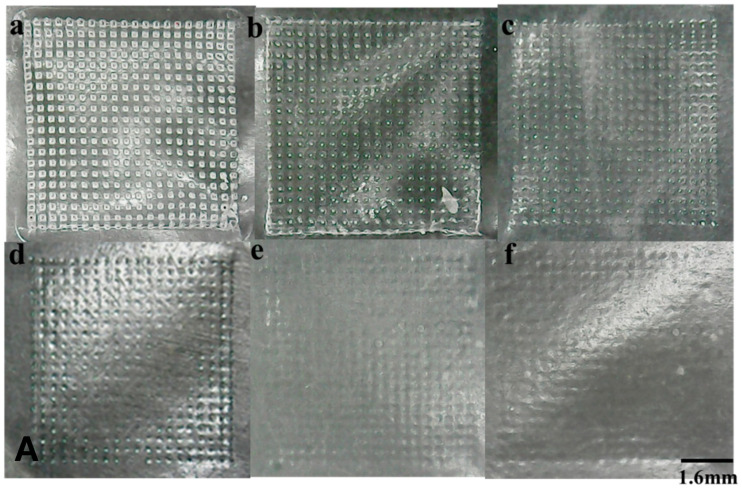

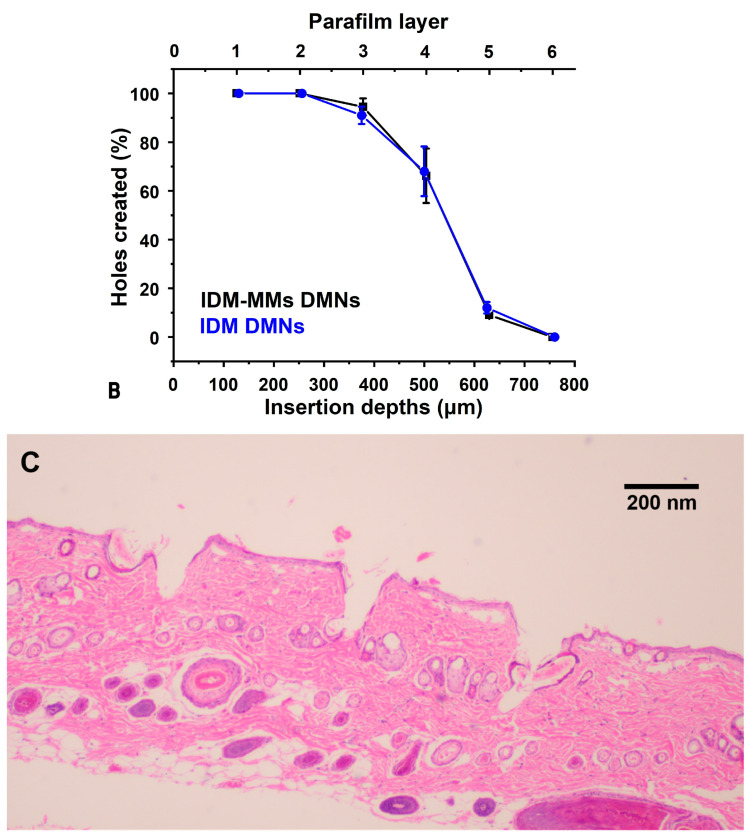
The micrographs of different layers of parafilm^®^ M after insertion by IDM-MMs-loaded DMNs (**A**). The first layer (**a**). The second layer (**b**). The third layer (**c**). The fourth layer (**d**). The fifth layer (**e**). The sixth layer (**f**); percentage of holes created in each parafilm layer by the IDM or IDM-MMs-loaded DMNs (mean ± SD, n = 3) (**B**); H&E-staining image after insertion of IDM-MMs-loaded DMNs into rat skin (**C**).

In order to deliver drugs into skin via DMNs, the shafts of DMNs should have enough mechanical strength and insertion ability to pierce the stratum corneum. The insertion force was set to 40 N, controlled by an analyzer. Figure 5A shows the holes created in each Parafilm^®^ M layer punctured by IDM-MMs-loaded DMNs. The quantities of holes formed in each layer were counted. Subsequently, the proportion of holes formed by the DMNs in relation to the depth was calculated, and it is shown in Figure 5B. Each PF layer has a thickness of around 126 μm [12]. As shown in Figure 5B, the first three layers were pierced by 100% of the total 400 MN shafts of IDM-MMs-loaded DMNs located on one array (depth, 378 μm). There was no significant difference observed in the insertion depth between the IDM and the IDM-MMs-loaded DMNs. This finding suggests that the IDM-MMs-loaded DMNs possess robust insertion capabilities. This might be owing to the small particle size of the MMs, since it was reported that the smaller the size of the particles loaded in DMNs, the less the effect on the mechanical strength of the DMNs [39]. Figure 5C shows a representative H&E staining slice, demonstrating that each needle could pierce the stratum corneum, the top layer of skin that needs to be penetrated to improve drug release.

### 3.5. In Vitro Release of IDM-MMs and IDM-MMs-Loaded DMNs

**Figure 6:**
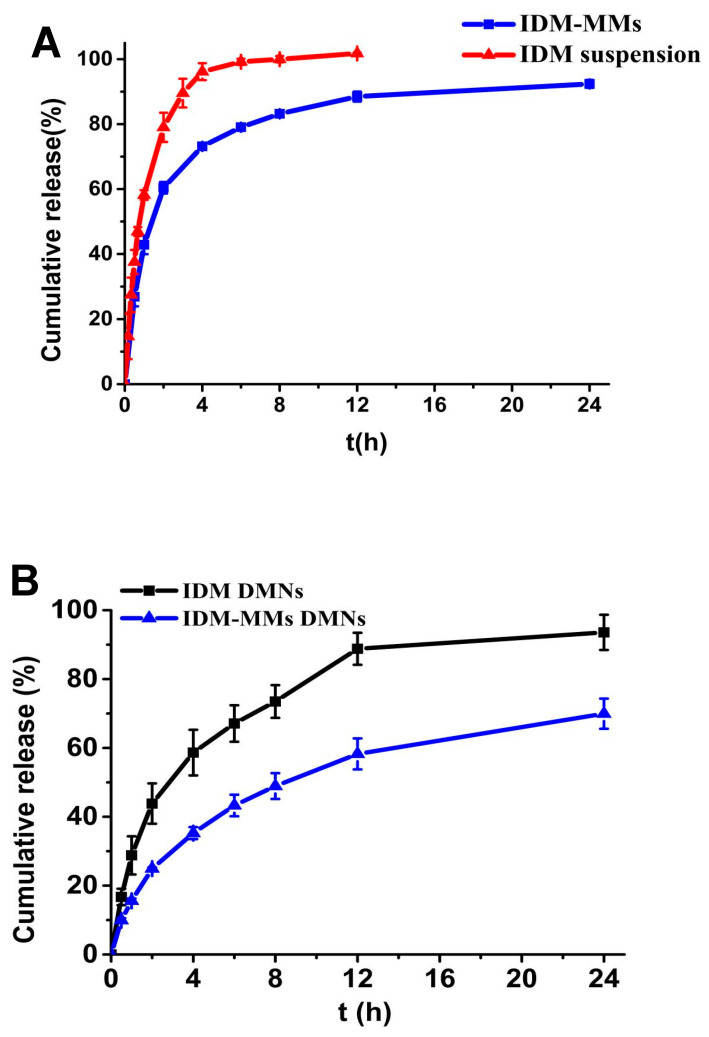
In vitro release profile of IDM-MMs (**A**); in vitro release profiles of IDM DMNs and IDM-MMs-loaded DMNs (**B**) (mean ± SD, n = 3).

Figure 6A shows the release behavior of the IDM-MMs. In comparison, the release profile of the free IDM exhibits a faster release rate than that of the IDM-MMs. There was an initial burst release in the first 2 h. This could have been the result of the IDM being absorbed on the surface of the MMs and then released in a typical sustained profile. To further describe the IDM release process, we assumed that the release profile of the IDM-MMs meets four frequently used models, including zero-order kinetics, first-order kinetics, and Higuchi and Ritger–Peppas equations. Furthermore, the first-order kinetics model exhibited the best fit with a highest correlation coefficient (R^2^ value) of 0.9808, indicating that the release behaviors of IDM-MMs may be controlled by both diffusion and erosion. The IDM dissolved in the disperse medium or adsorbed on the surface of IDM-MMs was firstly diffused to the receiving medium; and then, the IDM incorporated in IDM-MMs released slowly with the erosion of polymeric excipients [40].

The dissolution behavior of the IDM from the prepared IDM-MMs DMNs and IDM DMNs has been investigated during a 24 h period under physiological circumstances utilizing the dialysis bag method. The release profiles of IDM are shown in Figure 6B. Overall, in 24 h, less than 70% of IDM was released from the IDM-MMs DMNs, whereas more than 90% of IDM was released from the IDM DMNs. The initial burst release of the IDM-MMs DMNs was much slower than that of the IDM-DMNs (*p* < 0.05). Free IDM could be released rapidly from the DMNs through dissolution of the shaft by the release medium. In contrast, there might be two steps of IDM release from the IDM-MMs DMNs: firstly, the IDM-MMs are released from the DMNs through dissolution of the shaft; secondly, IDM is released from the IDM-MMs by polymer solubilization or diffusion via microchannels in the polymer matrix [41]. This indicated that the pre-formulation of IDM-MMs could not only increase the solubility of IDM, but also sustain its release from DMNs. By slowing down IDM release from DMNs, the mixed micelles may reduce medication fluctuations, perhaps leading to longer dose intervals and improved patient compliance [42].

### 3.6. In Vitro Transdermal Penetration of IDM-MMs-Loaded DMNs

**Figure 7:**
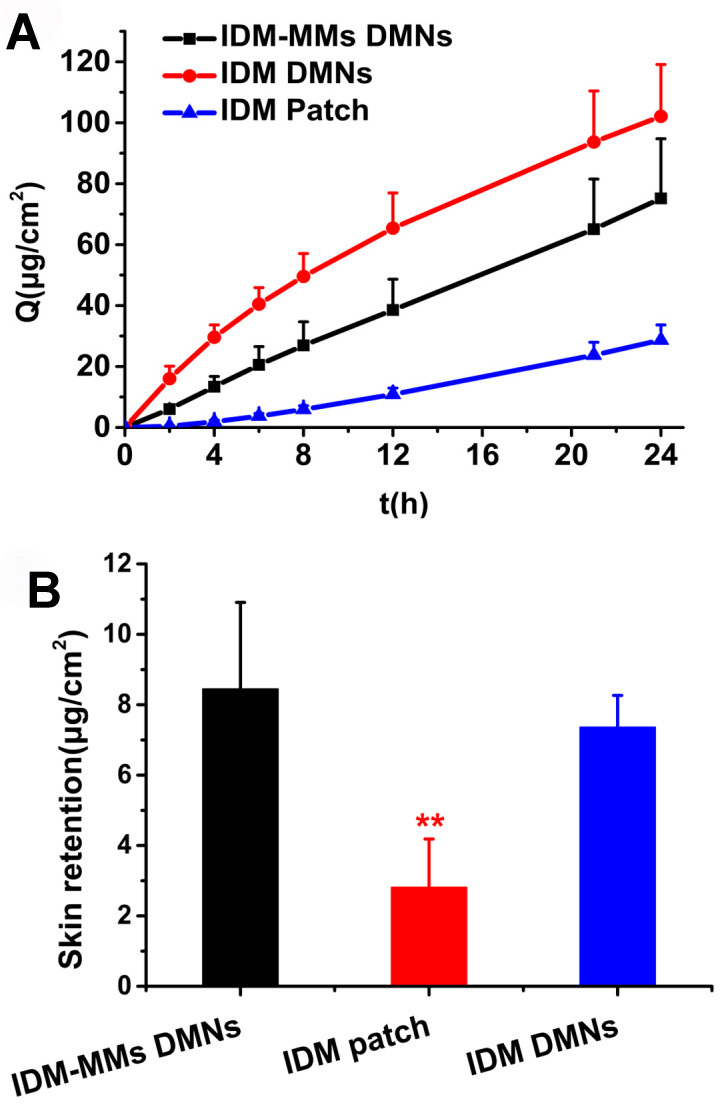
In vitro penetration results of IDM across full-thickness porcine skin (**A**); skin retention of IDM after transdermal penetration (**B**) (mean ± SD, n = 3. ** *p* < 0.01 compared with the IDM-MMs-loaded DMNs.

Figure 7A and Table 1 show the in vitro IDM penetration from the MMs DMNs, DMNs and commercial patch loaded with equal doses of IDM. The slope of the steady-state period of the cumulative penetration curves was used to determine the IDM steady-state flux (J_ss_). [43]. The cumulative penetration amounts of IDM in 24 h from the MMs DMNs and DMNs were 2.6 and 3.5 times higher than for the patch, respectively (*p* < 0.01). Moreover, the J_ss_ of the patch during the 8–24 h period was approximately 2-fold higher than the first 8 h, indicating a time lag in the penetration of IDM from the patch. The J_ss_ of DMNs was 6.18 μg·cm^−2^·h^−1^ in the first 8 h, and decreased to 3.25 μg·cm^−2^·h^−1^ during the remaining 16 h. In comparison, the J_ss_ of the MMs DMNs was 3.42 μg·cm^−2^·h^−1^ in the first 8 h, and 3.00 μg·cm^−2^·h^−1^ during the remaining 16 h, indicating a more sustained release of IDM transdermal penetration. These results were consistent with the in vitro release study. After insertion into the skin, the shafts of DMNs could be rapidly dissolved after contacting the skin interstitial fluid, which results in the liberation of the MMs, and then the MMs could diffuse into the deeper layers of skin and induce skin retention and sustained release [44].

The amount of IDM residue in the skin was also determined, and the results are illustrated in Figure 7B. The skin retention rates of IDM in the MMs DMNs group and DMNs group were approximately five times and three times higher than those for the IDM patch group, respectively. This indicated that the DMNs played an important role in enhancing skin retention. Moreover, the higher skin retention from the IDM-MMs DMNs compared to the IDM DMNs indicated the better retention capacity of the IDM-MMs. This suggested that, consistent with the cumulative penetration study, the MMs could function as a drug reservoir and sustain the release of IDM from DMNs.

### 3.7. In Vivo Pharmacokinetic Study

**Figure 8:**
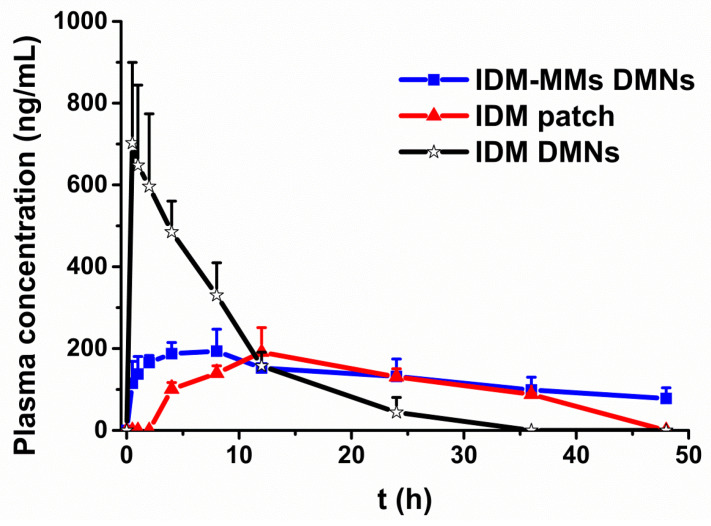
The mean plasma concentrations and time profiles of IDM DMNs, IDM MMs-loaded DMNs, and commercial patch (mean ± SD, n = 5).

We measured the plasma IDM concentrations following the treatment of rats with IDM-MMs DMNs, IDM DMNs and commercial patches, as shown in Figure 8. Although, menthol, one of the most commonly used penetration enhancers, was included in the commercial patch, the plasma IDM concentration was below the detection limit in the first 2 h in the patch group. The C_max_ was detected at 12 h as 190.92 ng/mL, and the IDM concentration was detectable until 48h. In contrast, the IDM DMNs group led to a much faster IDM absorption. T_max_ was only 0.5 h, and the C_max_ was 3.7 times higher than the patch group. These results indicated that the IDM penetration was improved by DMN administration in comparison to the commercial patch. Regarding the IDM-MMs-loaded DMNs, the plasma concentration was maintained at approximately 200 ng/mL for more than 12 h. Moreover, the plasma IDM was detectable even after 48 h. The results indicated that, consistent with the results of the in vitro penetration test, sustained IDM release could be obtained using the novel MMs DMNs system. This implied that the combination of MMs with DMNs is suitable for the delivery of drugs like IDM, which possess a narrow therapeutic window [4].

The pharmacokinetic parameters were calculated by DAS 2.0 and presented in Table 2. Although there was no significant difference between the peak plasma concentrations of the IDM patch and IDM-MMs-loaded DMNs, it should be noticed that the dose of IDM within the patch was much higher than those of the DMNs and MMs DMNs for the sensitivity of the HPLC detection. The relative bioavailability rates of the IDM DMNs and IDM-MMs-loaded DMNs compared to the IDM patch were 6.7 and 4.1, respectively, indicating that the bioavailability of the DMNs was higher than that of the commercial patch. This suggested that even though menthol was included in the patch as a penetration enhancer, both the penetration rate and the amount could be improved by DMNs. The relative bioavailability of the IDM DMNs was higher than the IDM-MMs-loaded DMNs. This might have been due to the fact that there was more drug loaded in the tip of IDM DMNs than for the IDM-MMs-loaded DMNs. The amount of drug loaded into the tips of the IDM-MMs DMNs was 29.2% of the total for the DMNs, and only half of that for the IDM DMNs. This might be because the IDM-MMs were more hydrophilic than the pure drug, which made them much easier to diffuse to the base of the DMNs during the drying process of preparation [45]. On the other hand, compared to the IDM DMNs, a lower C_max_ and longer T_max_ were obtained after the application of IDM-MMs-loaded DMNs, indicating that encapsulating IDM into MMs could result in a more sustained release.

### 3.8. Anti-Inflammatory Effect of IDM-MMs-Loaded DMNs

**Figure 9:**
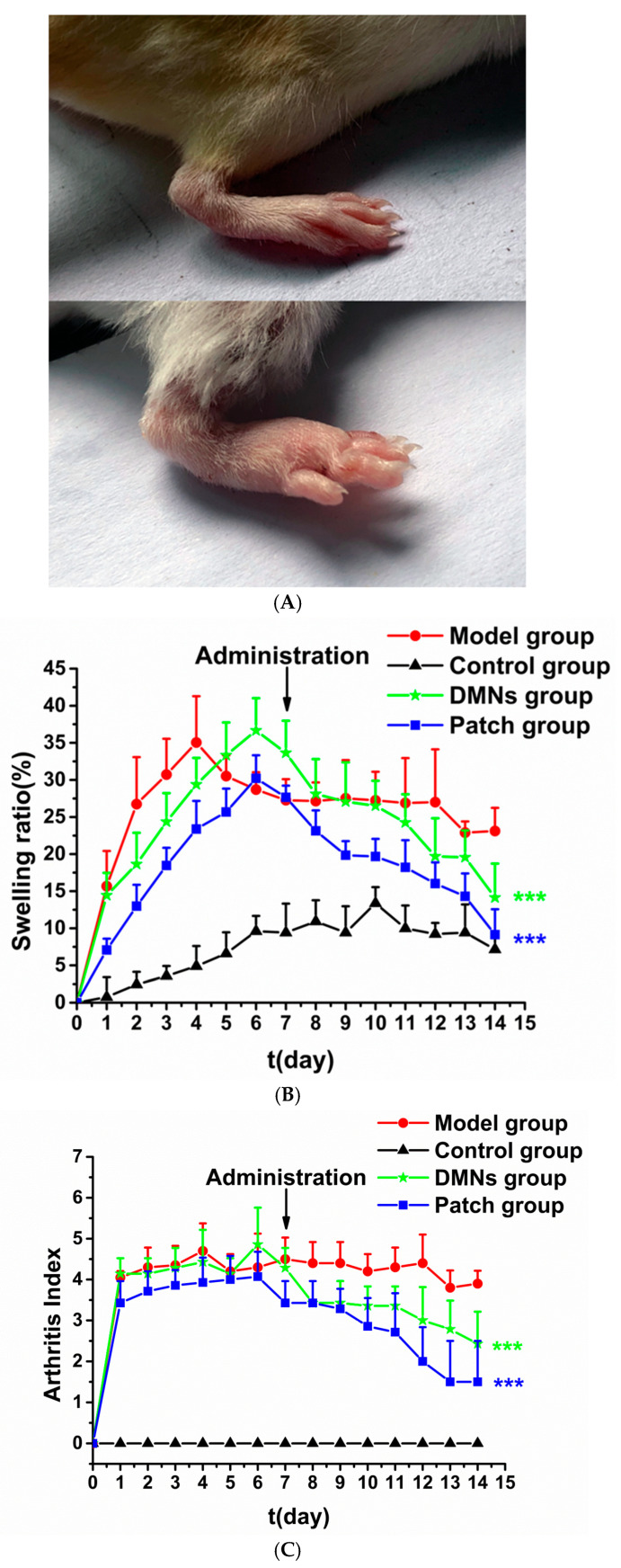

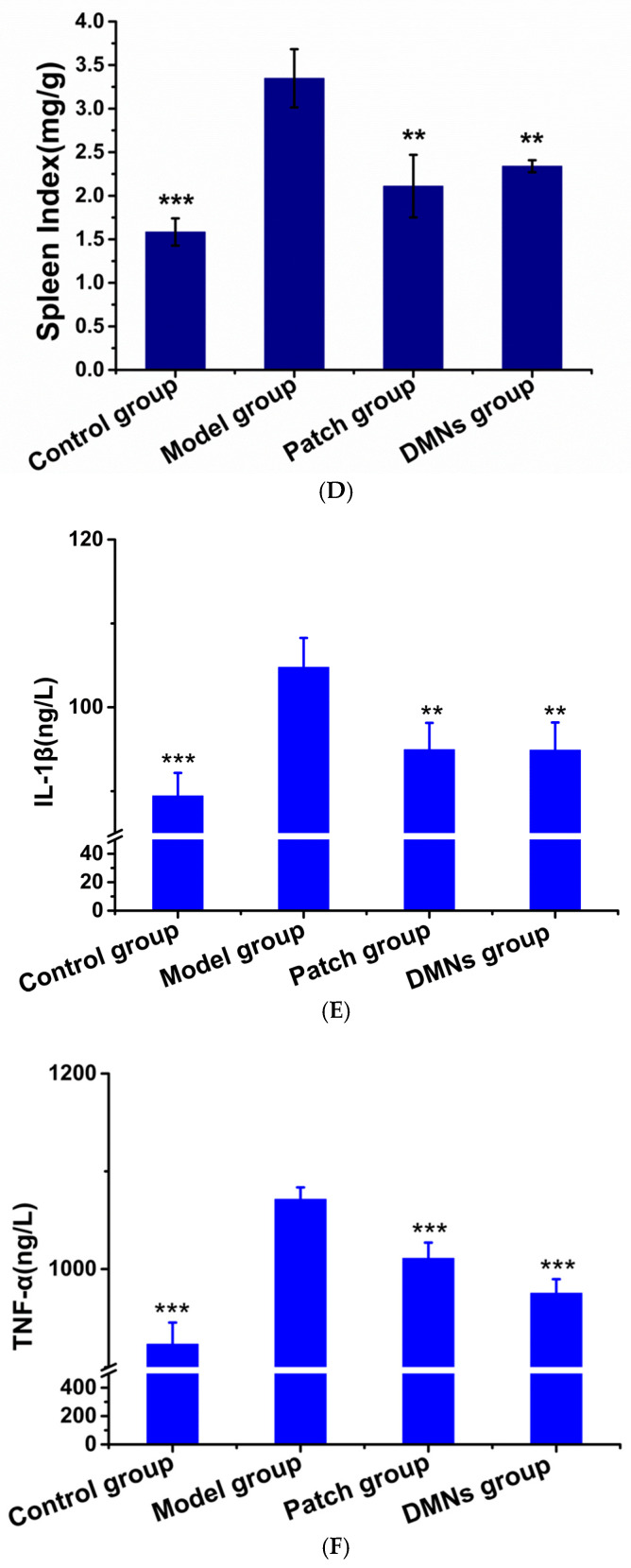
Representative photographs of mouse paw and joint before CFA induction (the picture above) and on day 7 after CFA induction (the picture below) (**A**); effect of IDM treatment on swelling ratio in AIA mice (**B**); arthritis index (**C**); spleen index (**D**); serum IL-1β level (**E**); serum TNF-α levels (**F**) (mean ± SD, n = 6. ** *p* < 0.01 compared with the AIA model group. *** *p* < 0.001 compared to the AIA. model group).

Adjuvant-induced arthritis (AIA) is a traditional rheumatoid arthritis animal model that is extensively used to evaluate anti-inflammatory drugs [38,46]. As shown in Figure 9A, the intraplantar injection of CFA caused significant paw swelling. Figure 9B,C shows the swelling ratio and arthritis index in different groups. Although the control mice’s paw circumference grew with body weight, their swelling ratio was much lower than that of the AIA mice (*p* < 0.001). As early as 24 h after injection, the swelling ratios and arthritis scores in the CFA-injected groups increased considerably compared to the control group. In the model group, the swelling ratio peaked on day 4 following adjuvant injection, and then fell slightly until day 7, and remained rather stable for the next 7 days. The arthritis scores of the model group were consistent from day 1 to day 14, indicating that the model was successfully established. After the IDM treatment, the swelling ratios in the patch and DMNs groups decreased from 27 ± 1% and 33 ± 4% (day 7) to 9 ± 3% and 13 ± 4% (day 14), respectively. The swelling ratio in both the patch and the DMNs groups fell by 18%. On day 14, the model group had a considerably higher swelling ratio than the patch and DMNs groups (*p* < 0.001). Consistent with the significant reduction in joint swelling, the arthritis index of the IDM patch and IDM-MMs-loaded DMNs decreased dramatically on day 14 (*p* < 0.001). The two treatment groups did not significantly differ from one another. (*p* > 0.05). Because the degree of joint inflammation and weight loss are closely related, we also looked into changes in the mice’s body weight. The results showed that the body weights of the mice gradually grew, with no significant difference seen between the four groups.

AIA may cause splenomegaly by increased β cell proliferation and immunoglobulin production [47,48]. As shown in Figure 9D, the spleen index of the AIA mice was approximately double that of the control mice, indicating spleen hyperplasia. Both the IDM patch and the DMNs treatment effectively reduced hyperplasia. The IDM-treated mice had a significantly lower spleen index compared to AIA mice (*p* < 0.01). IL-1β and TNF-α are key factors in the development of rheumatoid arthritis [49]. ELISA was used to determine the serum IL-1β and TNF-α levels in the AIA mice. Figure 9E,F demonstrates that the model group had considerably higher levels of IL-1β and TNF-α than the control group (*p* < 0.001). Compared to the model group, the administration of an IDM patch or IDM-MMs-loaded DMNs dramatically decreased the serum levels of IL-1β (*p* < 0.01) and TNF-α (*p* < 0.001). Treatment with IDM-MMs-loaded DMNs or an IDM patch reduced IL-1β and TNF-α levels similarly to the paw swelling and spleen index results. These results indicated that the IDM-MMs-loaded DMNs had a comparable reduction in arthritis severity to the IDM patch with the same dose and half the dose frequency. This might be because IDM-MMs-loaded DMNs can release for 48 h, as observed in the pharmacokinetic studies. The dose–effect relationship will be further investigated in future studies.

## 4. Conclusions

In this paper, the IDM-loaded MMs were prepared and combined with DMNs for the first time. The IDM-MMs showed small particle size, narrow distribution, and good stability. The IDM-MMs-loaded DMNs exhibited sufficient insertion capability to pierce the skin. In vitro drug release and transdermal penetration studies proved the sustained release and efficient skin penetration of IDM by the application of MMs-loaded DMNs. An in vivo pharmacokinetic study showed a steadier plasma concentration of IDM-MMs DMNs compared to free-drug-loaded DMNs. The relative bioavailability of the IDM-MMs DMNs compared to the commercial patch was 4.1. In AIA mice, IDM-MMs-loaded DMNs significantly reduced arthritis severity, including paw edema, arthritis index, spleen hyperplasia, and serum levels of IL-1β and TNF-α. In conclusion, the IDM-MMs-loaded DMNs fabricated in this study provide a potential route for the enhanced and sustained transdermal delivery of IDM.

## Figures and Tables

**Table 1:** Transdermal penetration parameters of IDM patch, IDM DMNs and IDM-MMs DMNs.

Group	J_0–8h_/μg·cm^−2^·h^−1^	J_8–24h_/μg·cm^−2^·h^−1^	Q/μg·cm^−2^
IDM-MMs DMNs	3.42	3.00	75.17 ± 19.59
IDM DMNs	6.18	3.25	102.08 ± 17.00
IDM patch	0.77	1.42	28.67 ± 4.98

**Table 2:** Pharmacokinetic parameters obtained after the administration of IDM-MMs DMNs, IDM DMNs, and commercial patch in rats (mean ± SD, n = 5).

Group	T_max_ (h)	C_max_ (ng/mL)	AUC_0–48h_ (μg/mL·h)	Frel
IDM-MMs DMNs	8	193.48 ± 53.57	6.12 ± 1.41	4.1
IDM DMNs	0.5	702.48 ± 197.31	5.51 ± 1.31	6.7
IDM patch	12	190.92 ± 60.03	4.12 ± 1.29	1

## Data Availability

The original contributions presented in this study are included in the article/Supplementary Materials. Further inquiries can be directed to the corresponding author.

## Supplementary Materials

The following supporting information can be downloaded at: https://www.mdpi.com/article/10.3390/pharmaceutics16121505/s1, Figure S1. FT-IR spectrogram of IDM; Poloxamer F127; Soluplus; physical mixtrure of IDM, poloxamer F127, and Soluplus; IDM-MMs. Figure S2. (A) HPLC chromatogram of IDM; (B) HPLC chromatogram of PVP/VA; (C) HPLC chromatogram of rat skin extracted solution. Figure S3. (A) HPLC chromatogram of blank plasma; (B) HPLC chromatogram of blank plasma + internal standard + IDM solution; (C) HPLC chromatogram of sample plasma + internal standard solution. Table S1. Accuracy, precision and recovery of IDM in HPLC analysis. Table S2. Stability of IDM in HPLC analysis. Table S3. Limit of f Quantification (LOQ) in IDM HPLC analysis from plasma. Table S4. Accuracy, precision and recovery of IDM in HPLC analysis from plasma. Table S5. IDM Stability in HPLC Analysis from Plasma.
