# Supplementary material for: Preparation and In Vitro/In Vivo Characterization of Mixed-Micelles-Loaded Dissolving Microneedles for Sustained Release of Indomethacin
Source: Pharmaceutics. 2024 Nov 22;16(12):1505. doi: 10.3390/pharmaceutics16121505 (PMC11728531; doi:10.3390/pharmaceutics16121505)
Supplement: Supplementary file 1 [file pharmaceutics-16-01505-s001.zip › pharmaceutics-3312035-supplementary.pdf]

Supplementary Information for

Preparation and In Vitro/In Vivo Characterization of Mixed-Micelles-Loaded Dissolving Microneedles for Sustained Release of Indomethacin

Baojie Wang [1,†], Langkun Liao [2,†], Huihui Liang [2], Jiaxin Chen [2] and Yuqin Qiu [2,3,*]

[1] The Third People's Hospital of Longgang District, Shenzhen 518112, China
[2] Department of Pharmaceutics, School of Pharmacy, Guangdong Pharmaceutical University, Guangzhou Higher Education Mega Center, 280 East Waihuan Road, Guangzhou 510006, China
[3] Guangdong Provincial Key Laboratory for Research and Evaluation of Pharmaceutical Preparations, Guangdong Provincial Engineering Center of Topical Precise Drug Delivery System, Guangdong Pharmaceutical University, Guangzhou 510006, China
* Correspondence: qiuyuqin@gdpu.edu.cn; Tel.: +86-20-39352117; Fax: +86-20-39352129
† These authors contributed equally to this work.

Table of the contents:

1. Preparation of mixed micelles
2. FT-IR analysis of IDM loaded mixed micelles
3. Methodology for validating HPLC analysis of IDM from *in vitro* penetration release media.
4. Methodology for validating HPLC analysis of IDM from plasma

1. Preparation of mixed micelles

Firstly, the ratios of Soluplus® and PF127 was optimized by the stability of mixed micelles. The micelles' stability improves as their critical micelle concentration (CMC) decreases. The CMC of micelles formed of Soluplus® and PF127 in a 4:1 weight ratio was found to be the lowest after measuring it in various weight ratios. Secondly, to optimize the IDM and carrier material ratios, the amount of IDM added to the micelles was gradually raised while the carrier material concentration remained constant, with micelle particle size and transparency used as assessment markers. When the particle size of the micelles increased dramatically and the transparency reduced significantly, the maximal drug loading capacity of IDM was reached. As a result, the weight ratio of IDM to carrier was determined to be 1:10 (w/w).

2. FT-IR analysis of IDM loaded mixed micelles

We have tried to investigate the physical state of IDM within the MM by FT-IR analysis. As it shown in Figure S1, IDM's characteristic peaks include 1717.62 cm^{-1}, 1692.03 cm^{-1} (C=O), 1589.90 cm^{-1} (C=C), and 1455.94 cm-1 (O-CH_3), etc. PF127's characteristic peaks include 3419.53 cm^{-1} (-O-H), 2883.85 cm^{-1} (-C-C stretching vibration), 1456.70 cm^{-1}, 1342.06 cm^{-1} (-C-H bending vibration), 1281.18 cm^{-1}, 1241.91 cm^{-1} (-C-C skeletal vibration), 1102.52 cm^{-1} (-C-O-C-). Soluplus's characteristic peaks include 3455.97 cm^{-1} (-O-H), 2860.89 cm^{-1} (-C-C stretching vibration), 1740.23 cm^{-1} (O=C-O-), 1638.16 cm^{-1} (O=C-N -), 1240.20 cm^{-1} (-C-C skeletal vibration), 1108.21 cm^{-1} (-C-O-C-. The characteristic peaks of the physical mixture spectrum are essentially a superposition of the IDM, PF127 and Soluplus characteristic peaks. The infrared spectrum of IDM-MMs is largely consistent with that of physical mixtures, but the -C=O peak of IDM and the - C-O-C- peak of the material have shifted to lower wavenumbers of 1681.98 cm^{-1} and 1096.24 cm^{-1}, respectively, indicating that the lone pair electrons on the material's ether bond combine with the carboxyl group of IDM to form hydrogen bonds. Other methods of analysis, like as XRD and DSC, could provide further information.

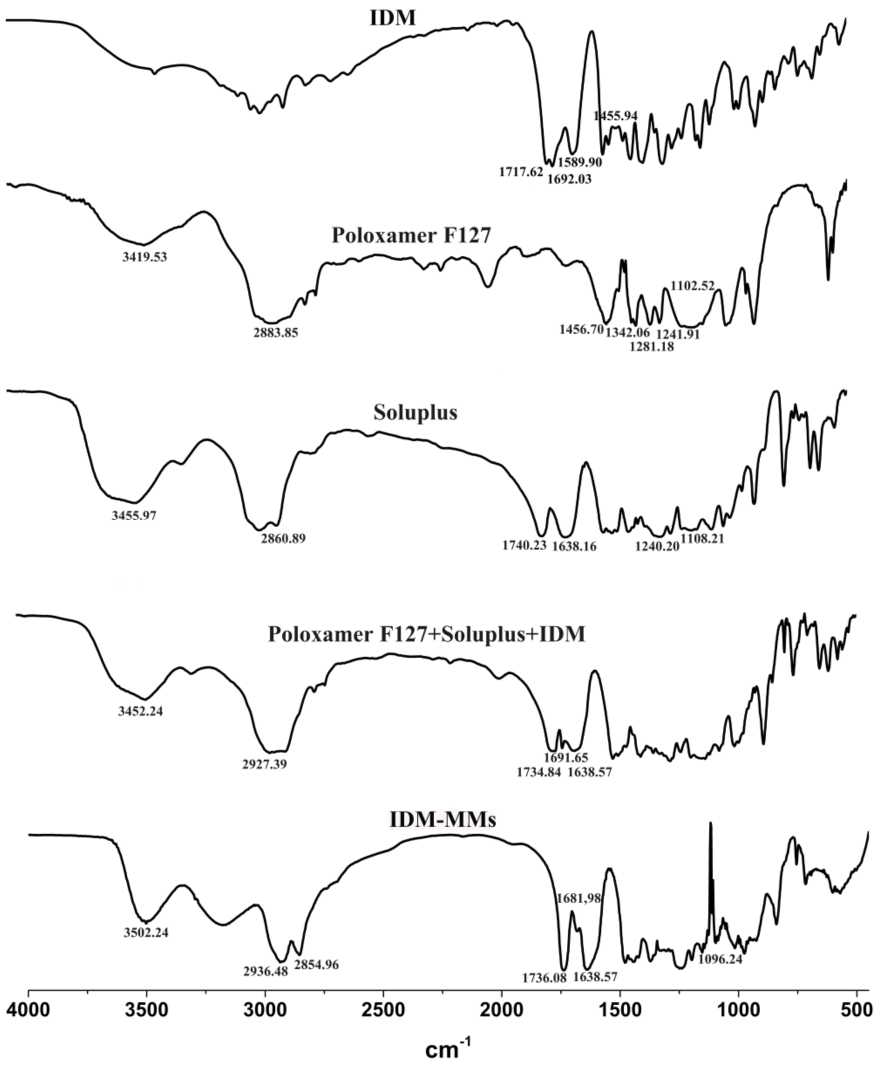

Figure S1. FT-IR spectrogram of IDM; Poloxamer F127; Soluplus; physical mixtrure of IDM, poloxamer F127, and Soluplus; IDM-MMs.

3. Methodology for validating HPLC analysis of IDM from *in vitro* skin penetration release media

3.1. Chromatographic conditions

IDM concentration was quantified using a HPLC system (LC-2010, Shimadzu, Japan). An ODS C18 column (250*4.6 mm, 5 μm, Dikma technology, China) was used with the mobile phase having a flow rate of 1.0 ml/min. The column temperature was set at 30 ºC. Acetonitrile-0.1mol/L ethylic acid in water (70:30 v/v) was the chromatographic condition employed. Detection of IDM was performed at a wavelength of 320 nm.

3.2. Linearity and range

Standard solutions of IDM were prepared at concentrations of 0.05, 0.25, 1, 4, 16, and 64 μg/mL. The linear relationship between the concentration of the standard solutions and the corresponding peak area was described by the equation y = 22.895x - 0.0335, with a correlation coefficient (r^2) of 1. This indicates a robust linear correlation for IDM within the concentration range of 0.05 to 64 μg/mL.

3.3. Specificity

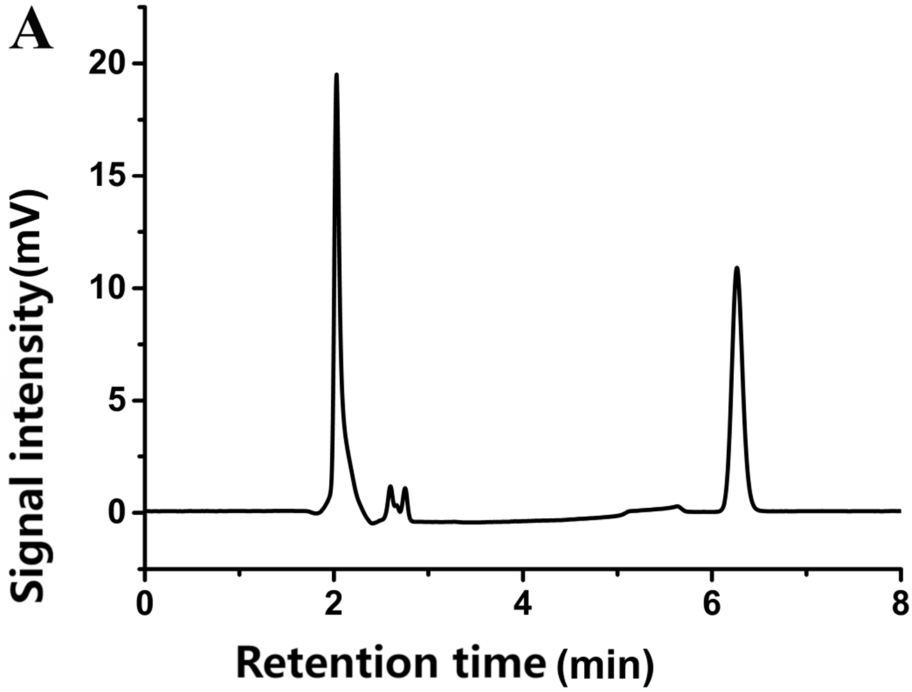

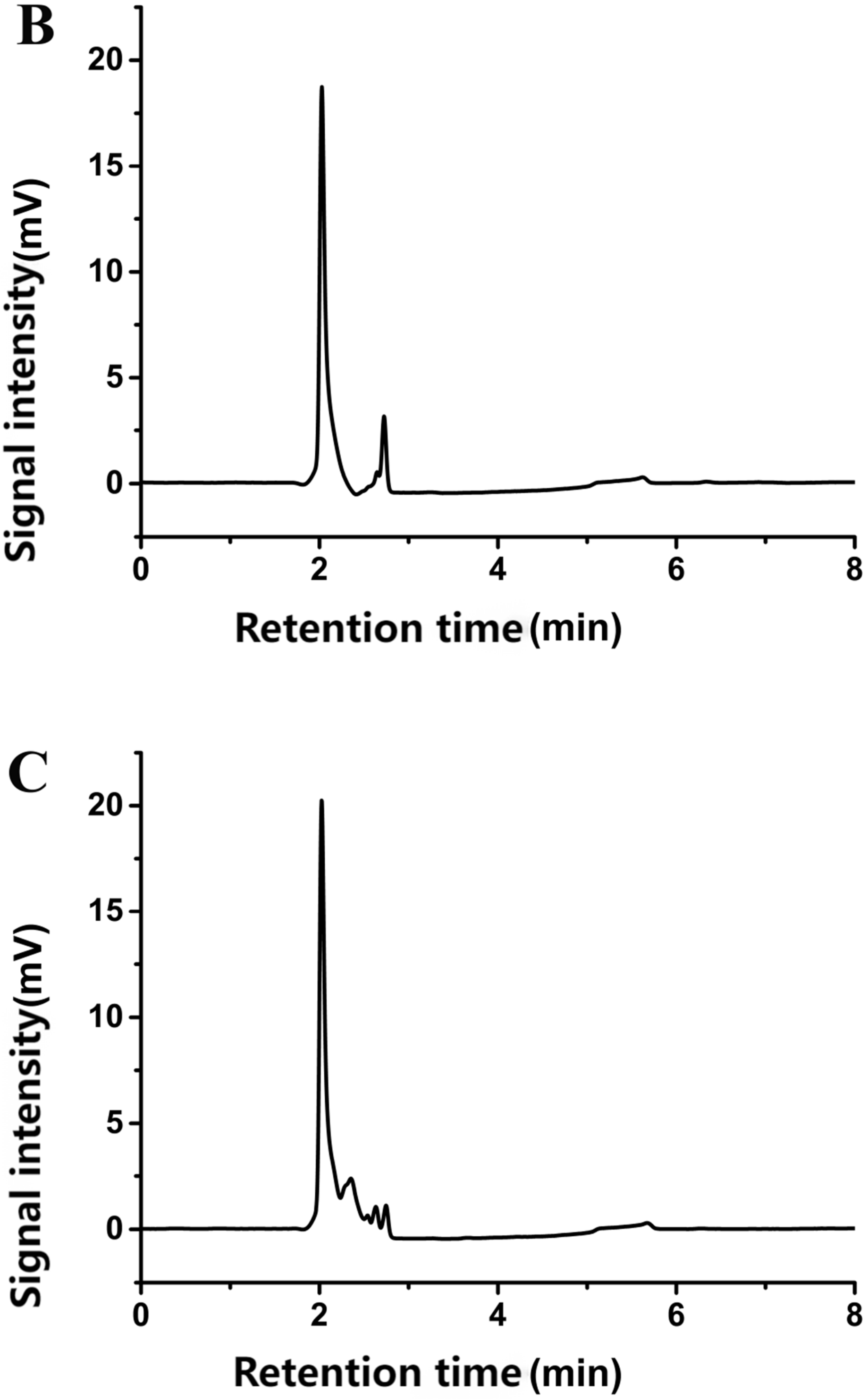

Figure S2. (A) HPLC chromatogram of IDM; (B) HPLC chromatogram of PVP/VA; (C) HPLC chromatogram of rat skin extracted solution

3.4. Accuracy, precision and recovery

Table S1. Accuracy, precision and recovery of IDM in HPLC analysis

IDM (μg/mL)	Accuracy (%)	Intra-day RSD (%)	Inter-day RSD (%)	Recovery (%)
0.25	101.37±1.01	1.72	1.71	102.24 ± 0.50
4	100.02±0.41	0.41	0.95	98.17 ± 0.57
64	100.89±0.33	0.33	0.49	100.88 ± 0.13

3.5. Stability

Table S2. Stability of IDM in HPLC analysis

IDM (μg/mL)	IDM (%)	
	0 h	24 h
0.25	100	102.29 ± 1.00
4	100	101.00 ± 0.41
64	100	99.19 ± 0.13

4. Methodology for validating HPLC analysis of IDM from plasma

4.1. Specificity

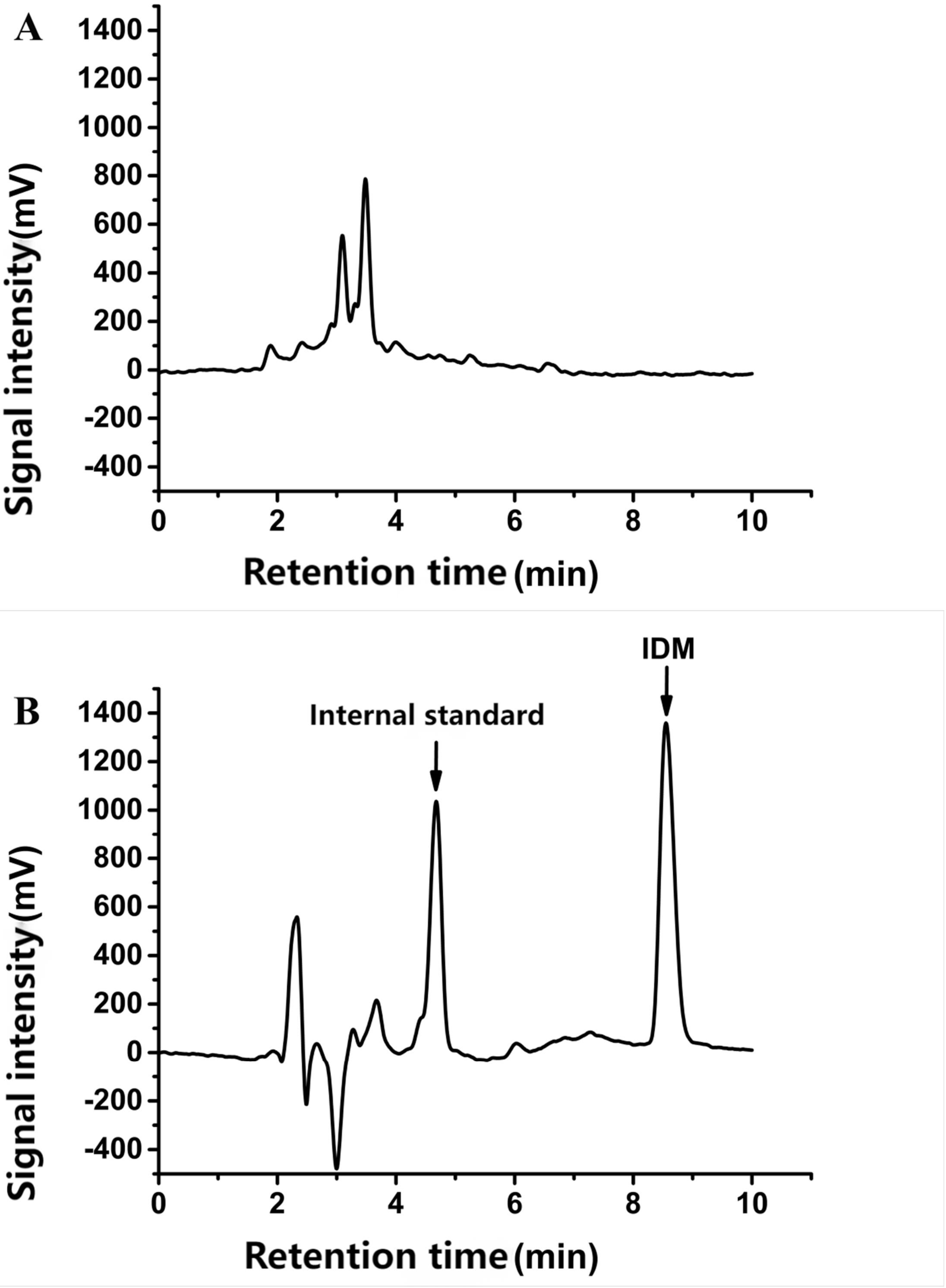

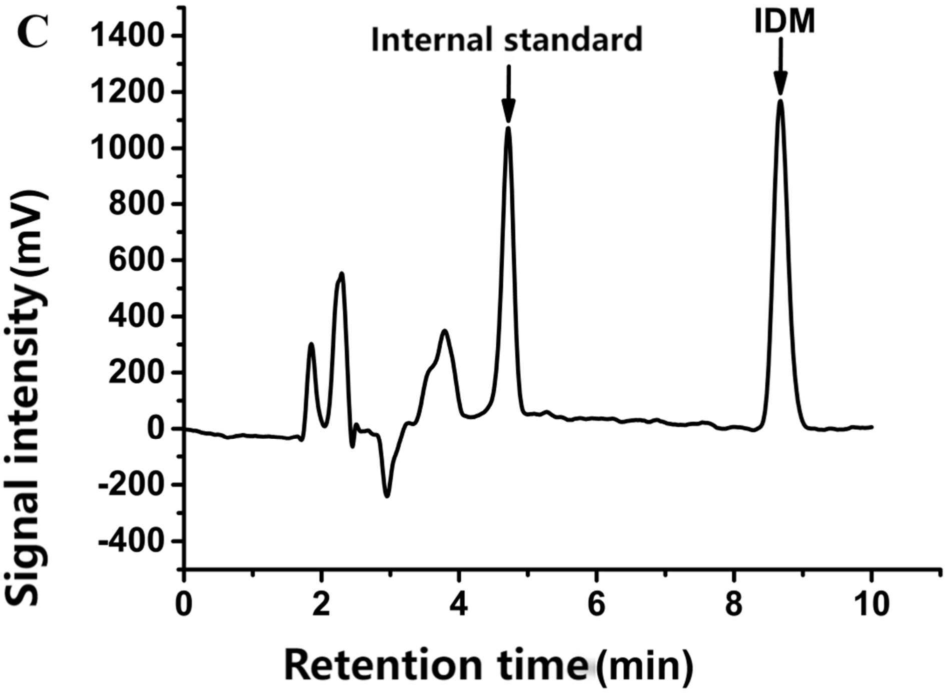

Figure S3. (A) HPLC chromatogram of blank plasma; (B) HPLC chromatogram of blank plasma + internal standard + IDM solution; (C) HPLC chromatogram of sample plasma + internal standard solution.

4.2. Linearity and range

The linear relationship between concentration and peak area for the standard plasma sample was described by the equation y = 4.2016x - 0.0248, with an R^2 value of 0.9998. This indicates a high degree of linearity for the analyte detection method (IDM) within the concentration range of 0.05 to 1.6 μg/mL.

2.2. Limit of Quantification (LOQ)

The concentration corresponding to a signal-to-noise ratio of 10 is defined as the limit of quantitation. In this study, when the standard plasma sample concentration was set at 0.05 μg/mL, the observed signal-to-noise ratio was approximately 10. To prepare standard plasma samples at this concentration, 180 μL of blank plasma was pipetted, followed by the addition of 20 μL of a 0.5 μg/mL standard solution and 20 μL of the internal standard solution. The results in Table S3 indicate that the accuracy of the measurements falls within the range of 80% to 120%, and the relative standard deviation (RSD) is maintained within ±20%. These findings confirm that the limit of quantification meets the established criteria.

Table S3. Limit of f Quantification (LOQ) in IDM HPLC analysis from plasma

Indicated value (μg/mL)	Measured value (μg/mL)	RSD (%)	Accuracy (%)
0.05	0.048	14.82	95.42 ± 14.14

4.3. Accuracy, precision and recovery

Table S4. Accuracy, precision and recovery of IDM in HPLC analysis from plasma

IDM (μg/mL)	Accuracy (%)	Intra-day RSD (%)	Inter-day RSD (%)	Recovery (%)
0.1	99.63 ± 1.53	1.54	1.54	69.56 ± 0.54
0.4	99.31 ± 2.01	2.02	2.56	62.48 ± 2.70
1.6	101.67 ± 1.41	1.38	2.16	61.50 ± 2.61

4.4. Stability

Table S5. IDM Stability in HPLC Analysis from Plasma

IDM (μg/mL)	IDM (%)		
	0 h	12 h	24 h
0.1	100	101.52 ± 1.16	99.36 ± 2.01
0.4	100	101.41 ± 0.99	101.57 ± 1.20
1.6	100	97.78 ± 1.46	98.83 ± 1.43
